# Grazer exclusion is associated with higher fast-cycling carbon pools but lower slow-cycling mineral-associated carbon across grasslands

**DOI:** 10.1073/pnas.2512048123

**Published:** 2026-02-02

**Authors:** Luhong Zhou, Shangshi Liu, Maarten Schrama, Deborah Ashworth, Richard D. Bardgett

**Affiliations:** ^a^Key Laboratory for Humid Subtropical Eco-geographical Processes of the Ministry of Education, School of Geographical Sciences, Fujian Normal University, Fuzhou 350117, China; ^b^Department of Earth and Environmental Sciences, The University of Manchester, Manchester M13 9PT, United Kingdom; ^c^Yale Center for Natural Carbon Capture, Yale University, New Haven, CT 06511; ^d^Yale School of the Environment, Yale University, New Haven, CT 06511; ^e^Institute of Environmental Sciences, Leiden Universiteit, Leiden 2333 CC, The Netherlands; ^f^Centre for Sustainable Soils, Lancaster Environment Centre, Lancaster University, Lancaster LA1 4YQ, United Kingdom

**Keywords:** climate mitigation, grassland management, mycorrhiza, nature-based solutions, soil carbon fractions

## Abstract

The removal of livestock grazers from historically grazed grasslands is widely proposed as a key strategy for the enhancement of soil organic carbon (SOC) for climate mitigation. Yet, accurate assessments of how grazer exclusion impacts SOC pools of differing stability are lacking, with most studies focusing on total SOC rather than the distribution of SOC within fast and more stable, slow-cycling pools. Here, we used 12 historically grazed grassland sites along an 800 km south–north gradient across the United Kingdom to test how particulate (POC) and mineral-associated organic carbon (MAOC) pools were linked to long-term (>10 y) exclusion of large domesticated grazers. We found that grazer exclusion was associated with relatively higher fast-cycling C pools, including plant and litter C, and to a lesser extent POC, but lower more stable, slow-cycling MAOC pools compared to grazed controls. Grazer exclusion was also associated with a marked shift in vegetation composition, with greater cover of ericoid mycorrhizal (ErM) shrubs over arbuscular mycorrhizal (AM) graminoids. This vegetation shift likely played a dual role in regulating SOC, contributing to higher POC via both the input of recalcitrant litter and by the enhancement of soil moisture and lower MAOC due to priming and decreased mineral protection of SOC. Our findings provide evidence that while the exclusion of grazers tends to favor fast-cycling C pools, it coincides with lower SOC persistence, potentially increasing the vulnerability of grassland SOC stocks to future climate change.

## Significance

The removal of livestock grazers from historically grazed grasslands is widely considered a beneficial strategy for restoring soil organic carbon (SOC) stocks and mitigating climate change. However, this view often overlooks the functional diversity of SOC pools and the contrasting mechanisms that govern their dynamics. Drawing on a network of long-term grazer exclusion experiments on grasslands, we show that grazer exclusion is associated with lower mineral-associated organic C—a key component of long-term C persistence—despite higher levels of faster-cycling, plant-derived pools. We also identify pathways that regulate these distinct SOC pools, which are linked to grazer-induced shifts in plant community composition. Our findings underscore the need to consider both the quantity and persistence of SOC in grassland management strategies.

Grasslands store over one-third of the global terrestrial ecosystem carbon (C) stock (1), with about 80% of this C being stored in soil. Global grasslands are primarily used for livestock grazing (2, 3), although historical overgrazing has led to widespread and substantial declines in soil organic C (SOC) storage (4), representing a significant C-cycle feedback to climate change and causing major reductions in ecosystem service supply (5). To address this, reduced livestock grazing, or the complete removal of grazers, has been proposed as a critical and scalable strategy for restoring degraded grasslands and rebuilding SOC for climate mitigation (2, 3, 6).

Previous studies have revealed highly variable outcomes of removing livestock grazers, such as cattle and sheep, from grasslands in the direction and magnitude of SOC change, depending on environmental context (7). Such inconsistencies may arise because SOC consists of different pools that may respond differently to grazing, as most previous studies treat SOC as a uniform entity. SOC can be conceptually categorized into particulate organic C (POC) and mineral-associated organic C (MAOC), each having different formation pathways and decomposition rates, and differing susceptibilities to land management (3). Mostly originating from plant polymers, POC persists in soil with a turnover time ranging from years to decades, due to its inherent biochemical recalcitrance and physical protection within aggregates (8). By comparison, MAOC, which is largely derived from root exudates and microbial necromass, and sorbs directly onto minerals such as iron–aluminum (Fe-Al) oxides (9, 10), is widely considered to be of critical importance for the long-term persistence of soil C over decades to millennia (11). Revealing the response of these functionally different C components to changes in grazing regimes is therefore crucial to ensure the long-term persistence of grassland soil C, yet it remains underexplored (12).

It has been proposed that grazing may have divergent impacts on soil POC and MAOC pools, but this remains largely untested in real-world settings (12) and the underpinning mechanisms remain poorly understood. Previous conceptual frameworks have been based on herbivore-mediated shifts in the quantity and quality of resources entering the soil as key regulators of SOC dynamics (13, 14). Evidence also points to a critical role for different plant functional types in SOC storage, resulting from differences in nutrient acquisition strategies that influence microbial metabolism (e.g., enzyme activities) and SOC decomposition via the priming effect (15–17). Specifically, herbaceous plants (most forbs and graminoids) typically form symbioses with arbuscular mycorrhiza (AM) fungi, exchanging photosynthesized C for improved inorganic nutrients and water uptake (18). By comparison, ericoid mycorrhiza (ErM) predominantly associated with roots of ericaceous shrubs and typically introduce complex organic compounds (e.g., chitin, cellulose, hemicellulose, pectin, and polyphenolics) into the soil (18, 19). Moreover, ErM plants possess the ability to degrade aromatic macromolecules through the action of extracellular enzymes including a range of hydrolytic and oxidative enzymes that are typically not produced by AM fungi (17, 20). This pathway is expected to lead to the accumulation of oxidative products, thus promoting SOC storage, particularly in soils that are rich in organic matter (21). As such, grazer-induced shifts in grassland vegetation composition, especially in the relative dominance of AM graminoids and ErM shrubs (6, 22), could play a significant, albeit poorly understood, role in regulating the size and persistence of SOC pools. Frameworks that incorporate interactions between vegetation shifts and microbial processes into SOC formation and persistence may help to better predict impacts of grazer exclusion on SOC storage and understand the underlying mechanisms involved.

Here, we assessed how the cessation of grazing by domestic herbivores impacts fast- and slow-cycling SOC pools and their persistence, and identified mechanisms involved. This was tested by sampling soils across twelve seminatural montane grassland sites along an 800 km north–south gradient in the United Kingdom, each with paired plots that were either grazed by sheep or had livestock excluded by fencing for >10 y. To identify associated mechanisms driving SOC storage and relative distribution of POC and MAOC pools in response to grazer exclusion, we evaluated a range of potential controlling factors, including plant mycorrhizal type, litter quality, soil geochemical properties (e.g., soil moisture, soil nitrogen, Fe-Al oxides), and microbial metabolism (e.g., hydrolytic and oxidative enzymes, and priming effect). Since greater dominance of ErM shrubs linked with grazer exclusion can lead to the production of larger quantities of recalcitrant litter, this could slow microbial decomposition and favor POC accumulation (8). Further, grazer exclusion can elevate soil moisture and consequently reduce reactive metal oxides in humid soils, processes that are not conducive to MAOC formation (23–25). Consequently, an increased accumulation of POC relative to MAOC could affect SOC persistence following grazing exclusion, due to shifts in litter quantity and quality, enzyme production, and soil abiotic conditions (8, 10, 17, 23, 26, 27). We hypothesized that: (1) grazer exclusion favors POC accumulation, but destabilizes MAOC, thereby reducing the persistence of SOC; (2) higher abundance of ErM plants associate with grazer exclusion regulate the distribution of POC and MAOC pools through contrasting pathways: POC is primarily controlled by induced changes in soil variables, such as soil nitrogen and moisture, while MAOC is mainly driven by Fe-Al oxides and microbial processes, such as the priming effects and microbial enzymatic activities. In this study, the effects of grazer exclusion are defined as the relative difference compared with the standing control level (i.e., plots under continued extensive sheep grazing maintained at typical stocking densities of 1 to 2 ewes per hectare per year) at the time of sampling, rather than as their temporal changes since the time of grazer exclusion, which would require baseline data for inference.

## Results

### Higher Aboveground Biomass and Restructured Plant Community in Grazer-Excluded Grasslands.

As expected, ungrazed grasslands consistently exhibited higher biomass and C content of aboveground vegetation by 0.91 kg m^−2^ and 21.12 g kg^−1^, respectively, and those of litter by 1.24 kg m^−2^ and 16.48 g kg^−1^, respectively, indicating higher aboveground C stocks compared with grazed plots (*P* < 0.001, Table 1 and *SI Appendix,* Fig. S1). There were no significant differences in slope (*P* = 0.57) or soil texture (*P* = 0.51) between grazed and ungrazed plots (*SI Appendix*, Table S1), thereby confirming that paired plots shared similar physical baseline conditions.

**Table 1.** Effects of grazer exclusion on aboveground and soil biogeochemical properties

Groups	Variables	Grazing	Grazer exclusion	*P* value
Plant properties	Organic layer (cm)	16.89 ± 1.81	21.22 ± 2.36	**<0.001**
	Aboveground biomass (kg m^−2^)	1.77 ± 0.14	2.67 ± 0.21	**<0.001**
	Vegetation C content (g kg^−1^)	453.34 ± 4.23	474.46 ± 4.15	**<0.001**
	Vegetation N content (g kg^−1^)	16.02 ± 4.10	15.91 ± 4.50	0.82
	Vegetation C:N	34.14 ± 1.11	36.08 ± 1.13	0.09
	Litter biomass (kg m^−2^)	1.14 ± 0.14	2.38 ± 0.21	**<0.001**
	Litter C content (g kg^−1^)	444.34 ± 5.12	460.82 ± 4.78	**<0.01**
	Litter N content (g kg^−1^)	15.72 ± 3.80	16.80 ± 4.00	**<0.01**
	Litter C:N	33.87 ± 0.98	32.82 ± 0.88	0.21
Soil properties	Moisture (g cm^−3^)	0.58 ± 0.02	0.61 ± 0.02	0.08
	pH	4.49 ± 0.09	4.44 ± 0.09	0.44
	Bulk density (g cm^−3^)	0.47 ± 0.05	0.44 ± 0.04	0.38
	fPOC (%)	71.36 ± 3.03	76.40 ± 2.53	0.051
	fMAOC (%)	28.64 ± 3.03	23.60 ± 2.53	0.051
	Available N (mg kg^−1^)	3.07 ± 0.42	4.39 ± 0.60	**0.03**
	Soil N (g kg^−1^)	12.85 ± 0.90	13.28 ± 0.95	0.58
	Soil C:N	21.06 ± 1.03	21.44 ± 1.08	0.50
	Fe_d_ (g kg^−1^)	2.37 ± 0.46	1.93± 0.50	**<0.001**
	Al_d_ (g kg^−1^)	0.55 ± 0.07	0.52 ± 0.14	0.18
	Al_d_ + 0.5Fe_d_ (g kg^−1^)	1.73 ± 0.29	1.48 ± 0.32	**0.01**
Microbial biomass	MBC (mg kg^−1^)	1,442.4 ± 139.03	1,296.85 ± 134.14	0.27
	MBN (mg kg^−1^)	339.13 ± 31.94	304.53 ± 32.66	0.33
	MBC:MBN	7.76 ± 1.67	9.25 ± 1.49	0.43
Enzyme	GLC (nmol h^−1^ g^−1^ soil)	2,383.63 ± 272.40	2,159.63 ± 200.47	0.46
	NAG (nmol h^−1^ g^−1^ soil)	620.48 ± 53.28	659.46 ± 68.14	0.58
	PHO (nmol h^−1^ g^−1^ soil)	6,9927.73 ± 7,559.67	73,096.13 ± 9,680.40	0.70
	PER (nmol h^−1^ g^−1^ soil)	6,633.72 ± 1,370.26	7,511.25 ± 1,427.01	0.32
	POX (nmol h^−1^ g^−1^ soil)	6,26.42 ± 109.43	1,179.00 ± 284.55	**0.04**
	Oxidases (nmol h^−1^ g^−1^ soil)	7,260.14 ± 1429.62	8,690.26 ± 1,489.28	0.09
	C use efficiency	0.27 ± 0.02	0.31 ± 0.02	0.06
Priming effect	PE_g_ (mg CO_2_-C g^−1^ POC)	13.38 ± 4.04	16.51 ± 2.94	0.52
	PE_c_ (mg CO_2_-C g^−1^ POC)	−0.67 ± 2.68	2.81 ± 1.15	0.24
	PE_g_ (mg CO_2_-C g^−1^ MAOC)	40.16 ± 7.62	64.70 ± 10.31	**<0.01**
	PE_c_ (mg CO_2_-C g^−1^ MAOC)	7.97 ± 7.43	13.40 ± 5.11	0.55

C, carbon; N, nitrogen; POC, particulate organic carbon; MAOC, mineral-associated organic carbon; fPOC, fraction of POC in total SOC; fMAOC, fraction of MAOC in total SOC; MBC, microbial biomass carbon; MBN, microbial biomass nitrogen; Al_d_+0.5Fe_d_, weight-normalized contents of Fe_d_ and Al_d_; GLC, β-glucosidase; NAG, N-acetyl glucosaminidase; PHO, phosphatase; POX, phenoloxidase; PER, peroxidase; PE_g_, glucose-induced priming effect; PE_c_, cellulose-induced priming effect.

**Fig. 1.**
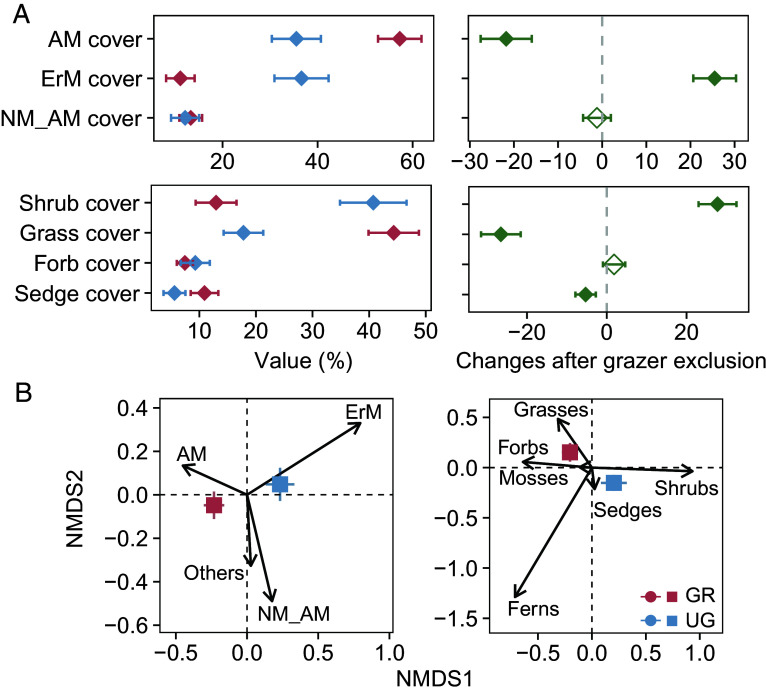
Differences in plant community composition between grazed (GR) and ungrazed plots (UG). (*A*) Cover of plant functional groups in grazed and ungrazed plots on the *Left*; and differences in functional groups associated with grazer exclusion are based on linear mixed models, with solid symbols indicating significant differences at *P* < 0.05, and hollow symbols indicating nonsignificant differences on the *Right*. (*B*) Nonmetric multidimensional scaling (NMDS) showed the differences in plant functional composition. The AM, NM_AM, and ErM in the figures represent arbuscular mycorrhizal, facultative arbuscular mycorrhizal, and ericoid mycorrhizal species, respectively.

Moreover, grazer exclusion corresponded with a marked shift in plant community composition (Fig. 1), with greater cover of dwarf-shrubs (by 27.77%, *P* < 0.001), but lower cover of grasses (by 26.55%, *P* < 0.001) and sedges (by 5.31%, *P* = 0.04) (Fig. 1*B*). When classified based on mycorrhizal functional types, ungrazed plots had lower cover of AM plants by 21.76% (*P* < 0.001) and greater cover of ErM plants by 25.49% across sites (*P* < 0.001, Fig. 1).

### Soil Mineral-Associated Organic Carbon (MAOC) Is Lower Under Grazer Exclusion.

**Fig. 2.**
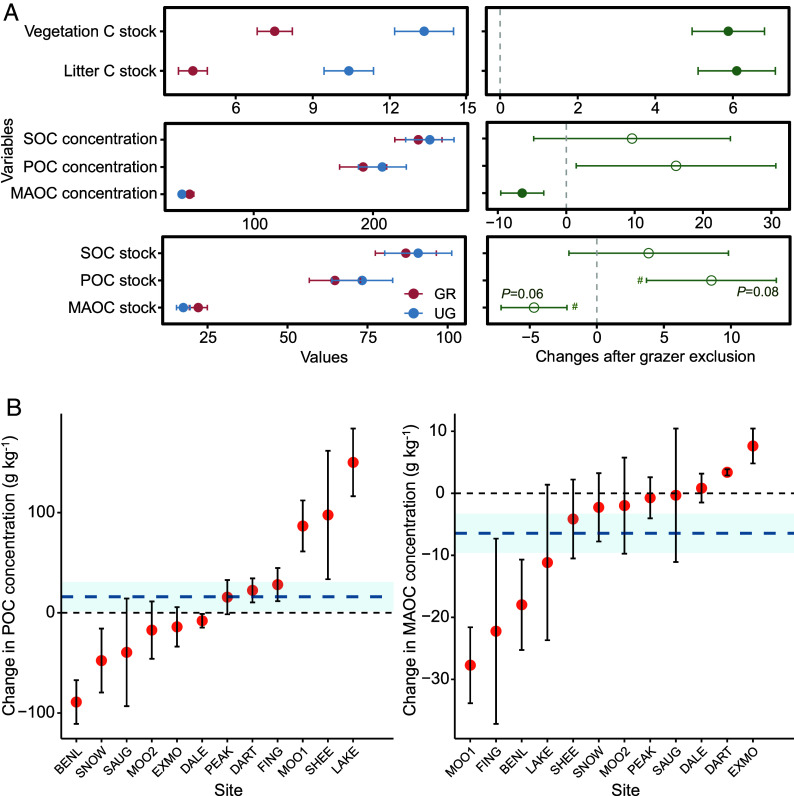
Differences in aboveground and soil carbon (C) pools in response to grazer exclusion. (*A*) patterns of C pools in grazed (GR) and ungrazed (UG) plots on the *Left*; and shifts of different C pools on the *Right*, with solid symbols indicating significant differences at *P* < 0.05 (n = 12). The units of C stocks and concentrations are Mg C ha^−1^ and g kg^−1^, respectively. The symbol “#” indicates the marginal significance at *P* < 0.1. (*B*) changes in particulate organic carbon (POC) and mineral-associated organic C (MAOC) in response to grazer exclusion across the twelve sites. Error bars indicate SE. Sites include Ben Lawers (BENL), Dartmoor (DART), Exmoor (EXMO), Glen Finglas (FING), Glensaugh (SAUG), Glenshee (SHEE), Lake District (LAKE), Moor House (MOO1), North Pennines (MOO2), Peak District (PEAK), Snowdonia (SNOW), and Yorkshire Dales (DALE).

While grazer exclusion did not significantly affect total SOC concentration (Fig. 2*A*), it did significantly alter the composition of SOC. Specifically, MAOC concentration was lower in ungrazed plots across sites by on average 6.44 g kg^−1^ (*P* < 0.05, Fig. 2*A*), and POC concentration was higher, albeit nonsignificantly (*P* = 0.28). Since soil bulk density did not differ significantly between grazed and ungrazed plots (Table 1), stocks of MAOC and POC followed patterns similar to their respective C concentrations (Fig. 2*A*), and the total SOC stock remained comparable (86.93 vs 90.80 Mg C ha^−1^, respectively).

Differences in C concentration of soil fractions across grazed and ungrazed plots showed considerable variation across sites, ranging from −88.99 to 150.15 g kg^−1^ for POC and −27.72 to 7.63 g kg^−1^ for MAOC (Fig. 2*B*). For example, although ungrazed plots showed lower and relatively similar MAOC in most sites, sites such as DART and EXMO exhibited modestly higher values, which underscores the context-dependent effects of grazer exclusion on soil C fractions.

Grazer exclusion was also associated with relatively higher soil available nitrogen concentration and phenoloxidase (POX) activity (both *P <* 0.05), but lower content of Fe-Al oxides (i.e., Fe_d_, and Al_d_+0.5Fe_d_ content; *P <* 0.01) across sites. Soil moisture (*P* = 0.08) and C use efficiency (*P =* 0.06) were marginally higher in grazer excluded plots (Table 1), and the glucose-induced priming effect was greater under grazer exclusion, particularly when normalized by MAOC across sites (*P <* 0.01). Moreover, the depth of the organic layer was on average 1.25 times deeper in ungrazed compared to grazed control plots (*P <* 0.001, Table 1).

### Factors Explaining Differences in Particulate Organic Carbon (POC) and MAOC Associated With Grazer Exclusion.

**Fig. 3.**
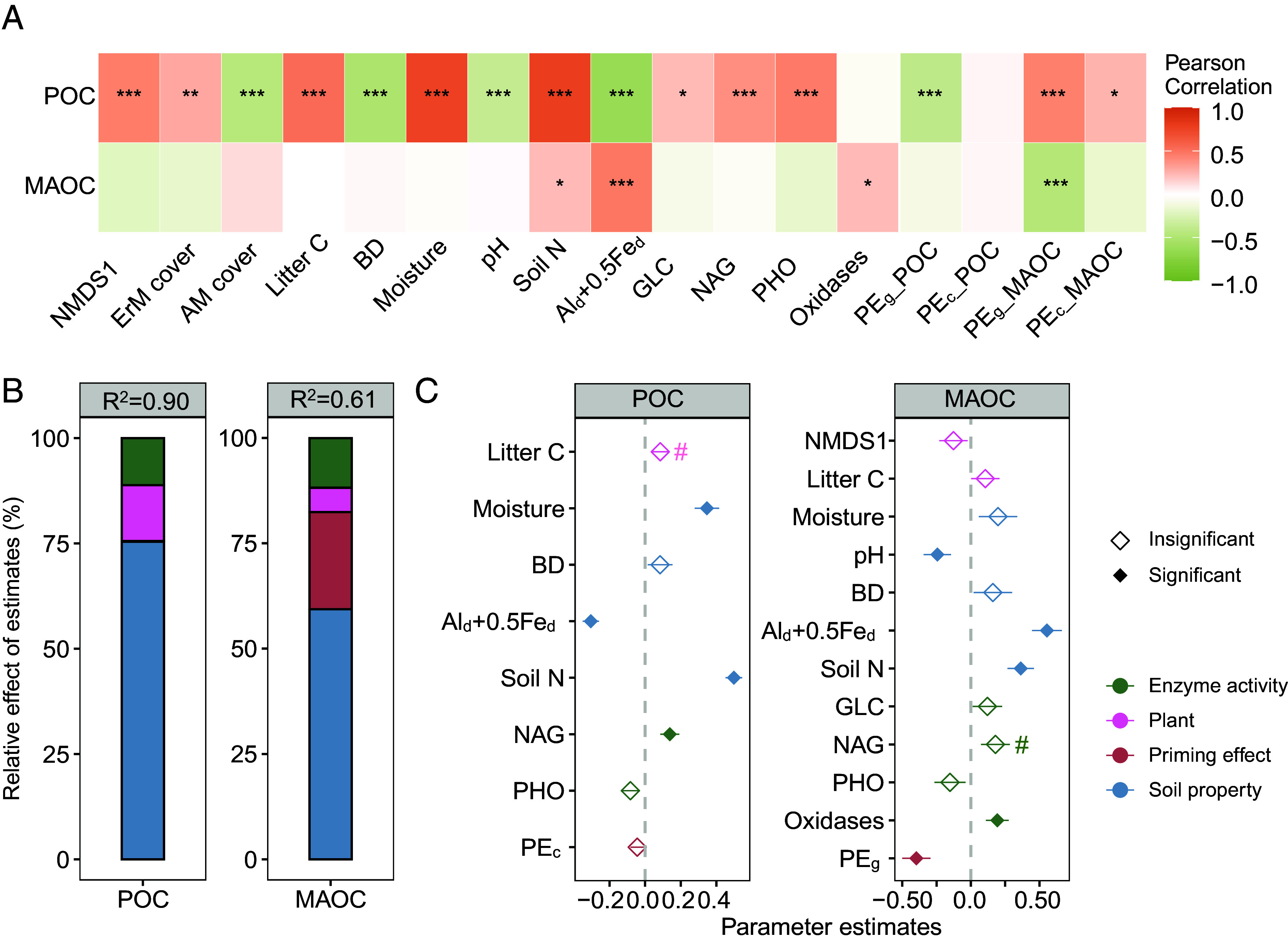
Effects of abiotic and biotic factors on soil POC and mineral-associated organic carbon (MAOC). (*A*) correlation of soil C components with the predictors. (*B*) the relative importance of the plant, enzyme, priming effect, and soil properties. (*C*) average parameter estimates of model predictors, and associated SE. The R^2^ of the averaged model and the *P* value are shown as ^#^*P* < 0.1; *P < 0.05; **P < 0.01; ***P < 0.001. NMDS1, the first axis of NMDS, shows the differences in plant functional composition; Al_d_ + 0.5Fe_d_, weight-normalized contents of Fe_d_ and Al_d_; GLC, β-glucosidase; NAG, N-acetyl glucosaminidase; PHO, phosphatase; PE_g_, glucose-induced priming effect; PE_c_, cellulose-induced priming effect.

We then identified the key factors explaining differences in SOC components associated with grazer exclusion (Fig. 3). For SOC components, the average models explained up to 90 and 61% of the total variation observed in POC and MAOC, respectively (Fig. 3*B*). Among the predictors, soil abiotic properties were the most important predictors in both models, explaining approximately 75.40 and 59.33% variation in POC and MAOC, respectively. The priming effect accounted for 23.04 and 0.12% of explained variance in MAOC and POC, respectively (Fig. 3*B*).

Among measured soil properties, soil nitrogen had a positive effect on the soil C components. Fe-Al oxides significantly and negatively affected POC, but positively affected MAOC. POC was found to be positively influenced by the N acquiring enzyme N-acetyl glucosaminidase (NAG) activity and litter C content, whereas MAOC was positively and significantly related to oxidase activity. Furthermore, the priming effect of glucose (normalized by C) significantly decreased MAOC (Fig. 3 *A* and *C*).

We found that the responses of POC and MAOC to grazer exclusion are predicted by different variables, suggesting that they are potentially driven by distinct mechanisms. Specifically, the effects of grazer exclusion on POC are best predicted by changes in soil nitrogen concentration, while differences in MAOC are best explained by variations in mineral protection capacity, measured as Fe-Al oxides, enzyme activity, and priming effects (*SI Appendix,* Fig. S2). Moreover, the effects of grazer exclusion were dependent on original soil C concentrations, as the differences in soil C concentrations were significantly decreased with higher soil C in the grazed controls (*SI Appendix,* Table S2). We found exclusion time did not significantly modify the effects of grazer exclusion on POC (*P* = 0.65) or MAOC (*P* = 0.37) (*SI Appendix,* Table S2 and Fig. S3).

**Fig. 4.**
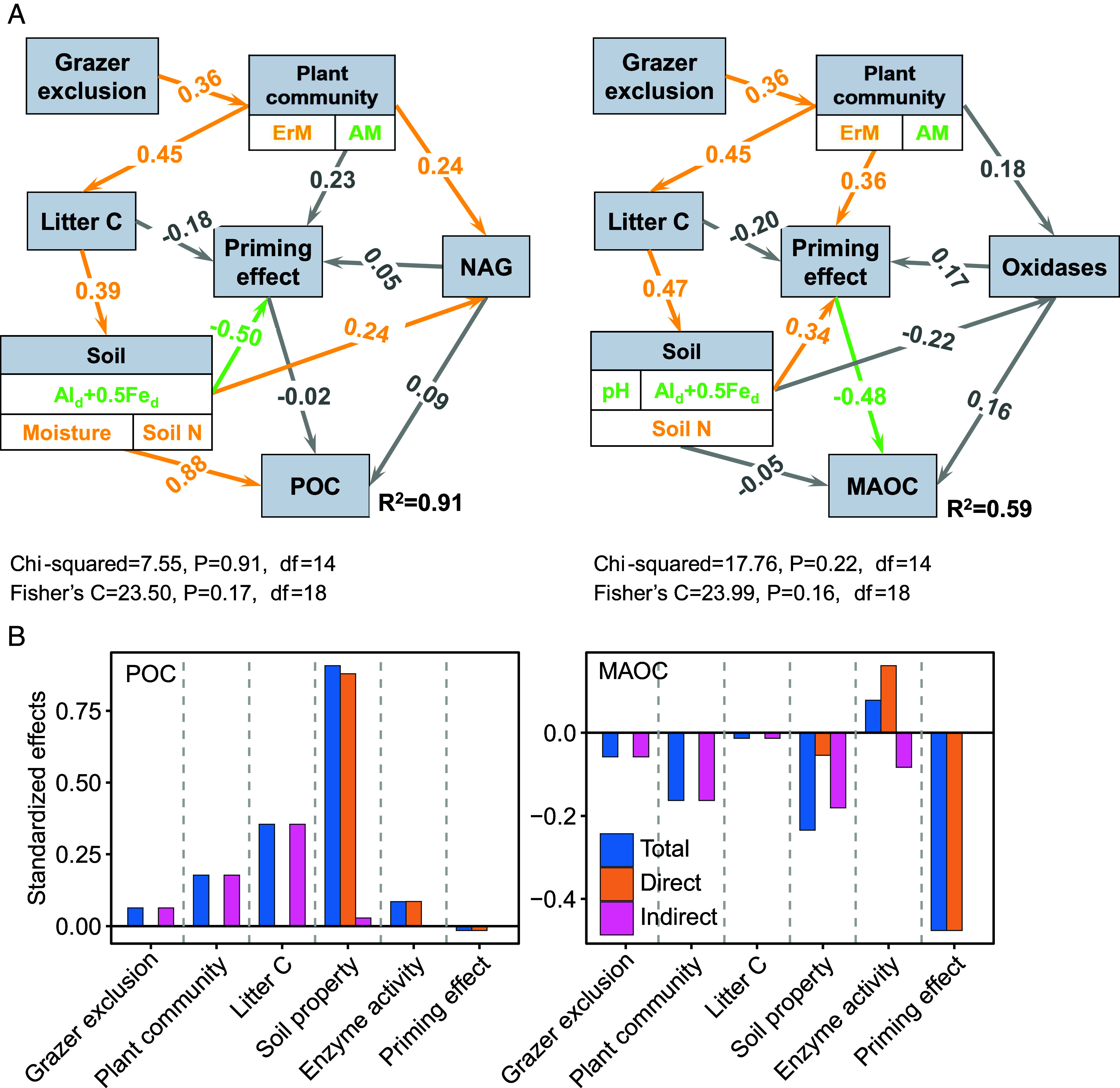
Effects of multiple drivers on SOC pools associated with grazer exclusion. (*A*) Structural equation modeling (SEM) analysis, nonsignificant paths are colored gray. Significant positive paths are colored orange, and negative paths are colored green. Numbers adjacent to arrows are indicative of the standardized effect size of the relationship. R^2^ denotes the proportion of variance explained. The significance levels for each predictor are *P* < 0.05. (*B*) bar graphs show the standardized effects of each variable of SEM. The positive or negative relationship of each variable in each group with the first axis of PCA was also indicated by arrows of different colors.

Finally, we used structural equation modeling (SEM) to further identify indirect effects of grazer exclusion on POC and MAOC (Fig. 4*A*). Consistent with the results of the model selection, soil nitrogen was the factor that most strongly and positively influenced POC, while mineral protection had the strongest and positive influence on MAOC. In comparison, MAOC was also negatively related to the priming effect, followed by soil properties whose contribution was mainly indirect (Fig. 4*B*). SEM also revealed that the effects of grazer exclusion on POC and MAOC are indirectly mediated by plant community composition, specifically the relative abundance of AM and ErM plants.

## Discussion

Based on data collected from a range of historically grazed seminatural grasslands, our study provides evidence that long-term grazer exclusion strongly influences the distribution of fast and slow C cycling pools with potential implications for SOC persistence. Our data show while long-term grazer exclusion plots contained more total ecosystem C and fast-cycling C pools, especially vegetation and litter C, and to some extent soil POC, it coincides with lower soil MAOC pools. Given that MAOC has a much longer mean residence time (from decades to centuries) than fast-cycling C pools (years to decades), this difference in MAOC with long-term grazer exclusion has implications for long-term SOC storage in grasslands, potentially increasing the susceptibility of SOC to future disturbances and climate change.

**Fig. 5.**
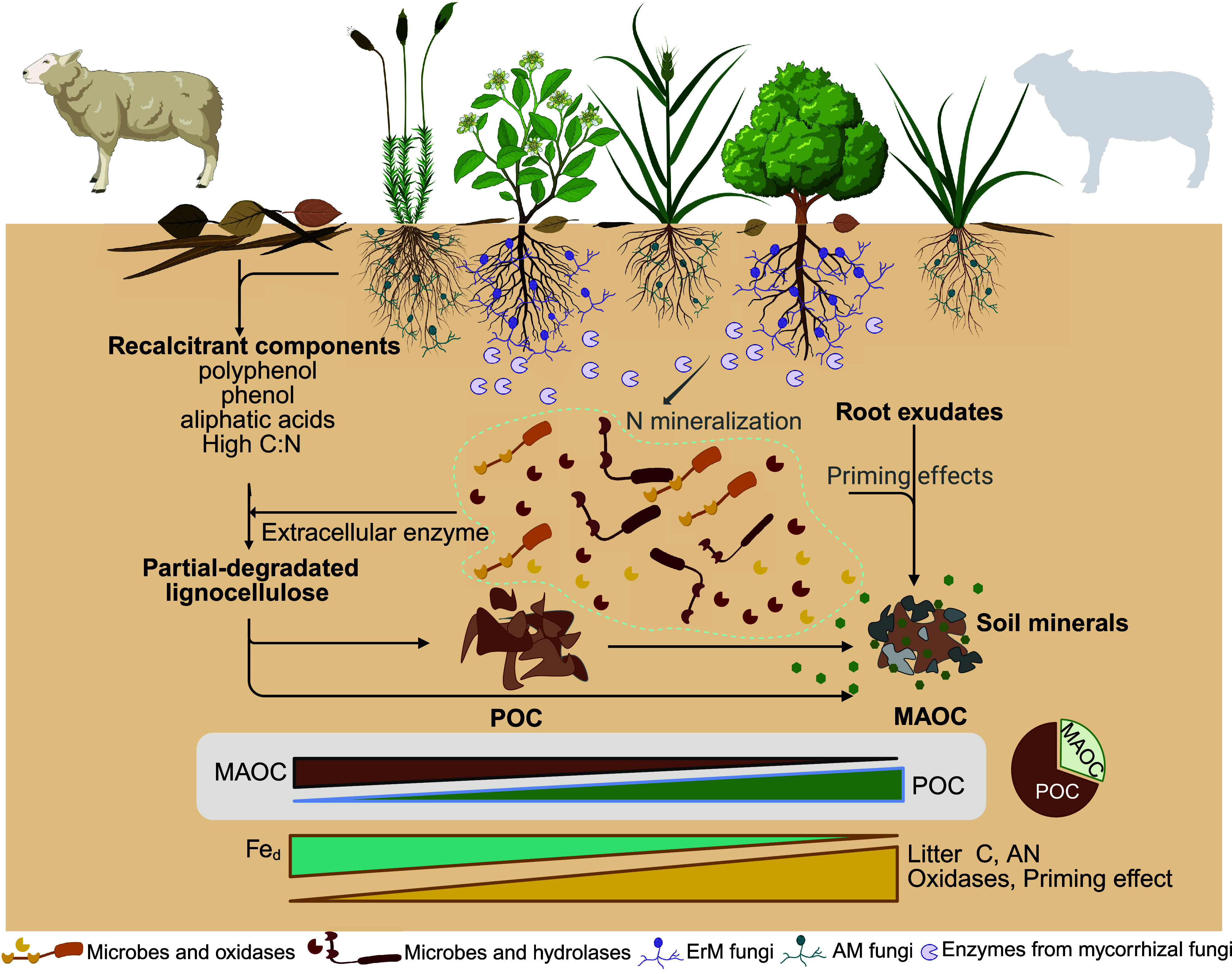
Key mechanisms by which grazing cessation influences soil organic carbon (SOC) pools. Grazing cessation leads to difference in litter quality, soil variables, and microbial processes, accompanied by a higher dominance of ericoid mycorrhizal shrubs. These differences in soil properties included a lower N limitation for the decomposition of recalcitrant litter, which primarily influences the accumulation of POC. In contrast, the lower MAOC in ungrazed grasslands was mainly driven by an enhanced priming effect through oxidases, with a lower mineral protection. Created with BioRender.com.

Multiple potential mechanisms might contribute to this shift in soil C persistence, but our data suggest that this change is primarily due to shifts in vegetation composition and mycorrhizal associations that result from exclusion of grazers (Fig. 5). As expected, grazer exclusion corresponded with notably higher fast-cycling C pools, particularly aboveground biomass and litter C, and, to some extent POC pool, depending on the standing control levels of each site (13). Our findings suggest that encroachment of ErM dwarf shrubs, coupled with a decrease in AM grasses, served as a major explanatory factor for the above response through changes in C input quality and associated microbial activities (6, 22, 28, 29). Such a vegetation shift associated with grazer exclusion aligns with previous studies, where litter accumulation suppresses grass growth, but facilitates shrub recruitment and growth by alleviating competition for soil water and nutrients (30–32). The AM plants (e.g., herbs) typically produce litters with high decomposability; by contrast, ErM litters are characterized by recalcitrant components such as polyphenolic and aliphatic compounds (33), suggesting the C input becomes relatively less degradable after grazing cessation. The Microbial Efficiency-Matrix Stabilization hypothesis also highlights that recalcitrant litter favors POC formation, while labile litter benefits MAOC formation (34). Our results validate this framework, revealing that the exclusion of grazing from historically grazed grasslands yields greater total ecosystem C stock, and is associated with more POC and lower MAOC, which is driven by higher cover of ErM shrubs over AM graminoids (10).

ErM shrubs also contribute more recalcitrant litter to the soil, influencing soil properties that predominantly drive POC accumulation (17, 35). The cessation of grazing is typically associated with higher soil moisture and changes nutrient availability, which significantly impact soil microbial communities and various functions such as C use efficiency and extracellular enzyme activities (10, 35–37). ErM fungi mining nutrients from SOM through enzymes provide soil inorganic N for saprotrophic microbes, prompting decomposers to preferentially extract nutrients from the labile components of recalcitrant litter, while resistant components such as polyphenols and aliphatic acids are preserved. The accumulation of undecomposed lignocellulose plant residues further supports POC accumulation, which is corroborated by the positive effect of litter C and NAG on POC in our models (38, 39) (Fig. 3). Specifically, shifts in POC linked to grazer exclusion were positively associated with changes in soil moisture and nitrogen, but negatively associated with soil minerals, which collectively explained the divergent responses observed across sites (*SI Appendix,* Fig. S2). For example, sites characterized by greater soil nitrogen and moisture but concurrent reactive minerals loss, such as DART, FING, LAKE, MOO1, PEAK, and SHEE, tended to exhibit higher POC in ungrazed plots. This mechanism of C accumulation may be particularly important in ecosystems with mineral-free surface soil horizons and high levels of oxidized organic matter with long mean residence times (21), which is typical of the seminatural montane grasslands studied here.

Despite the positive effect of grazer exclusion on fast-cycling C pools, our findings reveal a concurrent negative effect on slow-cycling MAOC, which represents the more stable fraction of soil C as it resists microbial decomposition and can persist in the soil for decades to centuries (8, 23). This lower MAOC associated with long-term grazer exclusion raises concerns, especially in light of current efforts to promote soil C sequestration as a climate change mitigation strategy. The underlying mechanism appears to involve both changes in microbial processes and a weakening of mineral protection. Microbial assimilation is hypothesized to regulate soil C dynamics through an “in vivo microbial turnover” pathway (40). In this process, soil microbes assimilate plant-derived C and convert it into various biomolecules, contributing to the MAOC pool (24, 41). The dominant mechanism posits that higher microbial C use efficiency is associated with greater accrual of MAOC, and therefore more persistence (34, 42). However, our findings do not support this hypothesis, as grazer exclusion coincided with lower MAOC but marginally higher C use efficiency (Table 1).

This contradiction suggests that MAOC formation may occur primarily through the alternative *“*ex vivo” pathway, where plant-derived complex components (e.g., lignin) are partially broken down by microbial extracellular enzymes into simpler monomers, which then associate with soil minerals to form MAOC (24, 35, 40). In ungrazed grasslands, the removal of labile nutrients from sheep dung and urine prolongs the litter-decomposition pathway by reducing microbial biomass (Table 1), which limits MAOC formation while promoting POC accumulation (13, 43). ErM shrubs can secrete hydrolases and oxidases to unlock organic nitrogen that is unavailable to AM fungi (15, 17, 44). A potential mechanism, the “Gadgil effect”, suggests that N competition by ErM fungi exacerbates N limitation for free-living decomposers. The competition is expected to reduce soil nitrogen mineralization and decomposition, leading to greater C (particularly POC) accrual under grazer exclusion (17, 45, 46). However, contrary to this expectation, we observed enhanced N mineralization into ammonium and nitrate in response to higher ErM cover in ungrazed grasslands, facilitated by activated NAG and oxidases (18, 47) (Table 1). Instead, our findings highlight the important role of priming effects in the dynamics of MAOC by alleviating the C and nitrogen limitation of free-living decomposers (16, 48, 49) (Fig. 3).

The shift in the dominance of mycorrhizal plant functional types associated with grazer exclusion may lead to a relative increase in root biomass and exudates, both of which can contribute to the priming effect (50, 51). Indeed, a recent meta-analysis revealed that the priming effect is more prevalent than the “Gadgil effect” in both AM and ErM systems (49). ErM fungi may induce priming by releasing plant-derived C and nonmelanized necromass into the soil, while also producing oxidative enzymes that depolymerize macromolecules (e.g., lignin), making the by-products available to free-living decomposers (17). Labile components of rhizodeposits (e.g., exudates and fungal mycelium) are expected to contribute primarily to the MAOC pool, whereas the complex components (e.g., sloughed-off cells and litter) are more likely to enrich the POC pool (34). MAOC has been considered less vulnerable to priming compared to POC due to mineral protection. However, recent studies suggested that living roots preferentially induce the MAOC decomposition by exuding organic acids to enhance microbial nitrogen availability, as MAOC exhibits a higher nitrogen mineralization potential than POC (47, 52, 53), due to the nitrogen-rich nature of MAOC compared to POC.

Our findings also indicate that the lower MAOC under grazer exclusion is likely mediated by significantly lower soil Fe-Al oxide content observed in these grasslands, which bind to organic C and form a major component of MAOC, especially in humid ecosystems with acidic soils, as studied here (9, 54). Globally, the metal-bound organic C constitutes nearly one-third of the total SOC stock and plays a crucial role in C stabilization (55). Despite the long residence time of Fe/Al-bound organic C, it is not permanent and can be destabilized by factors such as changes in water availability and soil pH, both of which may shift under grazer exclusion (56). For example, soil acidification can negatively affect microbial metabolism and mineral sorption, which promotes the dissolution of minerals and thereby declines the MAOC formation (57, 58). Root exudates, such as oxalic acid, chelate, and dissolve short-range minerals, while increased soil acidity enhances the solubility of Fe/Al oxides, transitioning them from solid minerals to soluble ions, thereby reducing effective soil binders and amplifying priming effects (48, 59). Moreover, the cessation of grazing might also reduce Fe/Al-oxides by altering soil aeration or redox potentials (60). Our findings reveal that the exclusion of grazers led to relatively higher litter C content, primarily explained by the expansion of ErM shrubs, which led to higher soil moisture levels across the study sites (Fig. 4). Such elevations in soil moisture, often associated with poor aeration in wet regions, can create a reductive environment that facilitates the reduction of Fe(III) to Fe(II), potentially release of Fe-bounded organic C (61). This phenomenon is supported by our findings, which show a negative relationship between soil moisture and Fe/Al oxides across our study sites (*SI Appendix,* Fig. S4). Given that grazer exclusion did not affect soil pH across our study sites, the lower concentration of Fe/Al oxides was more likely explained by the enhancement in soil moisture. This also explains the site-to-site variation in MAOC responses: sites characterized by higher soil moisture and lower Fe/Al oxides tended to exhibit lower MAOC, whereas well-aerated sites with stable Fe/Al oxides (e.g., DART, EXMO) showed slight higher MAOC in ungrazed grasslands. Collectively, our data suggest that the changes in MAOC associated with long-term grazer exclusion might be jointly mediated by a stimulation of C mineralization via priming effects and weaker mineral protection.

Overall, our findings reveal that long-term grazer exclusion has divergent effects on fast- and slow-cycling C pools across grasslands, positively affecting the total ecosystem C stock, particularly in the fast-cycling pools, but negatively affecting slow-cycling MAOC. We also show that the replacement of AM grasses by ErM shrubs plays a dual role in regulating soil C pools. These findings underscore the need to consider both plant functional types and mycorrhizal associations in C cycling models to improve predictions of C persistence under different management practices. Given that the ecological consequences of grazing management vary depending on climate, vegetation, soil context, and the regimes of grazing practices such as timing, frequency, and intensity (14), optimizing grazing management strategies should balance the potential trade-offs between fast-cycling C gains and long-term C persistence in a changing climate (7, 14). Additionally, since a large proportion of MAOC is found in the subsoil, MAOC loss induced by grazer removal may be substantially greater than estimated here. Future studies clearly need to incorporate subsoil layers to capture the whole-profile C changes, considering differences in root distribution, soil properties, and microbial metabolism (62). We also emphasize that the observed changes in plant and soil C pools under grazer exclusion represent relative differences compared with grazed controls at the time of sampling and should not be interpreted as directional shifts in ecosystem C stocks through time. For example, although grazing exclusion plots had higher levels of fast-cycling plant and soil C than grazed controls, this does not imply net C sequestration, as both treatments could have been experiencing losses, with ungrazed plots losing C at a slower rate. Future research would require repeat sampling to quantify the rates at which POC and MAOC change following grazer exclusion (63). Nevertheless, the divergent effects of grazer exclusion on POC and MAOC revealed in this study shed light on the distinct mechanisms of their formation, pointing to the need for targeted management strategies for soil-based C removal programs in grasslands.

## Materials and Methods

### Site Description and Sampling.

Prior to field sampling, we identified suitable sites across the main montane grassland regions in the United Kingdom. These sites represent a broad real-world ecological variability (e.g. climate, vegetation, and soil properties), allowing us to effectively test our hypotheses and generate broadly applicable insights into underlying mechanisms. All sites contained paired grazed and grazer-exclusion plots based on strict criteria to ensure consistency in land-use history and comparability across sites. Specifically, all sites 1) had never received inorganic fertilizers or herbicide applications prior to or following grazer exclusion; 2) were historically managed through extensive sheep grazing, and maintained at stocking densities of 1 to 2 ewes per hectare per year for grazed plots, which is typical of extensively grazed montane grasslands across the United Kingdom; and 3) had excluded grazers by fencing for over 10 y, with fencing replicated at least three times per site. In addition, sites were required to be at least 10 km apart to be considered independent from each other. Sampling was conducted from April to June 2015 across 12 seminatural montane grassland sites spanning an 800-kilometer south–north gradient (50.44 to 56.90°N, 1.68 to 4.45°W) of the United Kingdom, as described by Schrama et al. (64). This historical grazing, which is widespread across montane regions of the United Kingdom, has resulted in mosaics of vegetation, with patches of short and tall grass, and dwarf shrubs including *Vaccinium myrtillus, Calluna vulgaris, Erica cinerea, Empetrum nigrum, E. tetralix, and V. vitis-idaea.* Across sites, the time that grazers had been excluded by fencing ranged from 10 to 65 y.

At each site, we sampled paired 5 m × 5 m plots within four fenced (grazer exclusion) and adjacent unfenced, grazed areas (less than 10 m away), except at Exmoor, where we sampled three paired plots, and the Peak District, where we sampled six paired plots, totaling 98 plots, half grazed and half ungrazed, from 12 distinct sites. This paired-plot design also ensures comparability in terms of climate zone, vegetation type, topography, and soil properties baseline, factors known to strongly influence plant and soil properties (6, 7, 22). We established a 2 m × 2 m square quadrat in each plot, and plant species composition within each quadrat was recorded using a modified Braun–Blanquet method. All plant species present in each plot were listed, and their ground cover was estimated to the nearest 1%. Aboveground biomass and litter were collected from two 35 × 35 cm subplots within each plot. Additionally, four bulk density rings (100 cm^3^) were inserted in each plot to estimate bulk density. Soil stratification was also recorded in each plot. To reduce the effect of soil heterogeneity, ten cores (3 cm in diameter) of the upper 10 cm were randomly collected and then combined to form a single composite sample per plot. This composite sample was used for subsequent analyses of soil microbial communities, enzyme activities, priming effects, and physicochemical properties. To quantitatively verify the comparability of the paired grazed and ungrazed plots and rule out potential confounding factors, including inherent differences in soil conditions, we analyzed soil slope and texture (silt + clay percentages) for all pairs (*SI Appendix*, Table S1).

### Plant Mycorrhizal Functional Types.

Plant species were assigned to mycorrhizal types as follows: nonmycorrhizal-mycorrhizal (NM), obligate AM, facultative AM (NM_AM), EcM, ErM, orchid mycorrhizal (OrM), or nonidentified types according to the Harley and Harley database (65) and the FungalRoot database (66). Since EcM (*Quercus robur*) and OrM (*Dactylorhiza sp.* and *Orchis sp.*) plants were only found once, we combined these types with nonidentified types into the category “Others” for subsequent analysis.

### Quantification of Soil Organic C Components.

A size-based fractionation approach was used to separate the POC and MAOC fractions (23). In short, 30 mL of deionized water was added to 10 g of air-dried soil, which had been sieved to 2 mm, and a sonication energy of 150 J mL^−1^ was applied for dispersion. The dispersed soil was then thoroughly rinsed through a 53 µm sieve to separate the POC (>53 µm) from the MAOC (<53 µm) fractions using a wet sieving system with sieves and pans on a shaker (8, 67). Subsequently, the isolated fractions were oven-dried at 60 °C to a constant weight and then weighed. The C and N concentrations of each fraction were determined on an automated elemental analyzer (Vario EL Cube, Elementar Analysen systeme GmbH, Germany). Due to the variety of existing soil fractionation protocols, the measured POC and MAOC should be considered operationally defined by the specific method employed.

### Priming Effects.

First, 5 g soil was preincubated for one week in a 250-mL glass jar at 15 °C and 70% water holding capacity to activate microorganisms and minimize the pulse effect (68). Following preincubation, 3 atom% ^13^C-labeled glucose solution (Cambridge Isotope Laboratories, Inc.) and cellulose suspension (SCRSTANDARD, Shanghai) were added to simulate labile root exudates and litter or root inputs, respectively. The rate of C addition was set to 1% of the SOC concentration, according to previous studies (69, 70) (0.5 to 3.5%). An equivalent volume of deionized water was added to the control jars. Three additional empty jars were set simultaneously as blanks. A 20 ml bottle of 1 M NaOH solution was placed in each jar to trap CO_2_. All jars were incubated for 30 d at 15 °C, the average temperature throughout the study area. This 30-day incubation period allowed us to observe real priming effects rather than apparent priming (i.e., the turnover of microbial biomass), which typically occurs within the first 1 to 2 wk of incubation (69, 71). The NaOH solution was analyzed for total inorganic C using a TOC analyzer (Aurora 1030, OI analytical) and was analyzed for δ^13^C using ring-down cavity spectroscopy with an Automate Module (Picarro G2131-i Analyzer, Picarro Inc.). Priming effects were calculated using the following formulas

[1] $$f_{\mathrm{substrate}}=(\delta^{13}C_{\mathrm{total}}-\delta^{13}C_{\mathrm{control}})/(\delta^{13}C_{\mathrm{substrate}}-\delta^{13}C_{\mathrm{SOM}}),$$

[2] $$f_{\mathrm{substrate}}+f_{\mathrm{SOM}}=1,$$

[3] $$\mathrm{Priming}\;\mathrm{effect}=C_{\mathrm{total}}\times f_{\mathrm{SOM}}-C_{\mathrm{control}},$$

where *δ^13^C_total_*, *δ^13^C_control_*, *δ^13^C_substrate_*, and *δ^13^C_SOM_* represent the δ^13^C values of CO_2_ from the substrate-addition soil (glucose and cellulose, respectively), control soil, added substrate (glucose and cellulose, respectively) and soil organic matter (SOM), respectively. *C_total_* and *C_control_* are the total CO_2_-C from the substrate-addition soil and control soil, respectively.

### Plant and Soil Properties.

Vegetation and litter biomass were oven-dried at 70 °C for 48 h and weighed. Plant and soil C and nitrogen concentrations were quantified using an automated elemental analyzer (Vario EL Cube, Elementar AnalysenSysteme GmbH, Germany). The bulk density and moisture were measured after drying the soil at 105 °C for 72 h. Coarse fragments (e.g., gravel or rocks) were removed and accounted for in bulk density estimation, although sampled soils contained minimal coarse fragments. Soil texture was measured using the hydrometer method (25). Soil pH (1:2.5, soil-to-water ratio) was determined using a pH meter (Mettler Toledo, UK), and extractable nitrogen was measured using an autoanalyzer (Seal AA3, SEAL Analytical, UK). The microbial biomass C (MBC) and nitrogen (MBN) were quantified using the chloroform fumigation method (72). Dithionite-extractable iron (Fe_d_) and aluminum (Al_d_) which primarily represent both short-range order and crystalline phases, were extracted using the citrate-bicarbonate-dithionite (CBD) method (54, 73). Specifically, 0.25 g of soil was extracted using a mixed solution of 0.1 M sodium dithionite, 0.27 M trisodium citrate, and 0.11 M sodium bicarbonate, and continuously stirred in an 80 °C water bath for 15 min (61). The extracting suspension was then filtered through 0.45 μm membranes, and the Fe_d_ and Al_d_ were quantified using an inductively coupled plasma-optical emission spectrometer (ICP-OES, ICAP 6300, Thermo Scientific, USA). We then used weight-normalized contents of Fe_d_ and Al_d_ (Al_d_+0.5Fe_d_) to represent the Fe/Al oxide contents in the soil (54).

### Enzyme Assay.

*β*-glucosidase (GLC), *N*-acetyl glucosaminidase (NAG), and phosphatase (PHO) that catalyze key C-, nitrogen-, and phosphorus-cycling processes were measured photometrically using specific substrate analogues linked to specific pNPs. Briefly, 3.75 g of soil was suspended in 5 mL of 50 mM sodium acetate buffer at pH 5.0. Next, 150 µL of the soil slurry was combined with 150 µL of a specific substrate solution: 25 mM *p*NP-β-glucopyranoside for GLC, 5 mM *p*NP-N-acetyl-*β*-D-glucosaminide for NAG, and 5 mM *p*NP-phosphate disodium salt hexahydrate for PHO. Each sample was tested with four replicates on plates and incubated at 18 °C for different durations under continuous shaking: 1.5 h for GLC, 3.5 h for NAG, and 0.5 h for PHO. Following incubation, the blocks were centrifuged at 2,900 g for 5 min, and 100 µL of the supernatant was subsequently pipetted to transparent 96-well plates and mixed with 200 µL of 50 mM NaOH solution. Absorbance was measured at 405 nm using a plate reader (EZ400 Research, Biochrom, Germany). Oxidases including phenoloxidase (POX) and peroxidase (PER) enzyme activities were determined colorimetrically using L-3,4-dihydroxyphenylalanine (DOPA) as a substrate. POX and PER activities were measured at an absorbance of 450 nm using the same plate reader as above after incubation for 18.5 h and 20 h in the dark, respectively (74).

### Statistical Analyses.

We used linear mixed-effects models to evaluate impacts of grazer exclusion on the following response variables: slope of plots, aboveground and below-ground C pools (e.g., vegetation, litter, SOC, POC, and MAOC), plant community (e.g., cover), microbial properties (e.g., biomass, extracellular enzyme activities, priming effects, and C use efficiency), and soil geochemical properties (e.g., texture, pH, bulk density, moisture, Fe_d_, Al_d_, and nutrients). Grazer exclusion was used as a fixed factor, and the site/block was treated as a random factor in linear mixed-effects models using the “nlme” package (75). The distance matrices of plant mycorrhizal or functional composition between grazing and grazing cessation sites were visualized by nonmetric multidimensional scaling (NMDS) using the “vegan” package (76). The key predictors of the C components and persistence (POC and MAOC) were identified by a model selection method based on Akaike’s information criterion (77, 78). Variables were Z-score standardized before entered into the models, and variables with variance inflation factors more than 10 were removed due to high collinearity (79). The estimate for each predictor was calculated and the relative importance of plant properties, microbial enzyme activities, priming effects, and physicochemical properties in driving changes in soil C components and persistence was examined by an analysis similar to variation partition analysis based on the model averaging as mentioned above (78). Furthermore, we used SEM to examine the total, direct, and indirect relationships among grazing cessation, plant mycorrhizal functional composition, litter C, microbial enzyme activities, priming effect, and various soil biotic properties that influence soil C components based on an a priori model. Before constructing SEMs, we conducted a principal component analysis (PCA) to derive multivariate functional indices for soil properties. Initially, we simplified the models by removing nonsignificant pathways to improve the fit of the model, evaluated using Fisher’s C statistic, chi-square, and associated *P*-values. A good fit was indicated by *P* > 0.05. The SEMs were performed using the “piecewiseSEM” (80) and “lme4” (81) packages, with all regression models fitted using the *lme* function. The power analyses were conducted using the “simr” package (82), which indicated that our study design, with a sample size of 12 sites and replication within each site, can reliably detect medium to large effects (Cohen’s d = 0.5 to 0.8), although it has limited power for detecting small effects (Cohen’s d = 0.2), which would require inclusion of additional experimental sites and/or increased within-site sample size (*SI Appendix*, Table S3). All analyses were performed in R (83).

## Supplementary Material

Appendix 01 (PDF)

## Data Availability

Soil and plant variables data have been deposited in Figshare (84).
