# Supplementary material for: Grazer exclusion is associated with higher fast-cycling carbon pools but lower slow-cycling mineral-associated carbon across grasslands
Source: Proc Natl Acad Sci U S A. 2026 Feb 2;123(6):e2512048123. doi: 10.1073/pnas.2512048123 (PMC12890883; doi:10.1073/pnas.2512048123)
Supplement: Supplementary file 1 — Appendix 01 (PDF) [file pnas.2512048123.sapp.pdf]

PNAS

Supporting Information for

Grazer exclusion is associated with higher fast-cycling carbon pools but lower slow-cycling mineral-associated carbon across grasslands

Luhong Zhou[a,b], Shangshi Liu[b,c,d*], Maarten Schrama[b,e], Deborah Ashworth[b], Richard D. Bardgett[b,f]

[a] Key Laboratory for Humid Subtropical Eco-geographical Processes of the Ministry of Education, School of Geographical Sciences, Fujian Normal University, Fuzhou 350117, China

[b] Department of Earth and Environmental Sciences, The University of Manchester, Manchester, M13 9PT, United Kingdom

[c] Yale Center for Natural Carbon Capture, Yale University, New Haven, CT 06511

[d] Yale School of the Environment, Yale University, New Haven, CT 06511

[e] Institute of Environmental Sciences, Leiden Universiteit, Einsteinweg 2, Leiden 2333 CC, The Netherlands

[f] Centre for Sustainable Soils, Lancaster Environment Centre, Lancaster University, Lancaster, LA1 4YQ, United Kingdom.

*Corresponding author: shangshi.liu@yale.edu, or liushangshi@gmail.com

This PDF file includes:

Figures S1 to S4
Tables S1 to S3

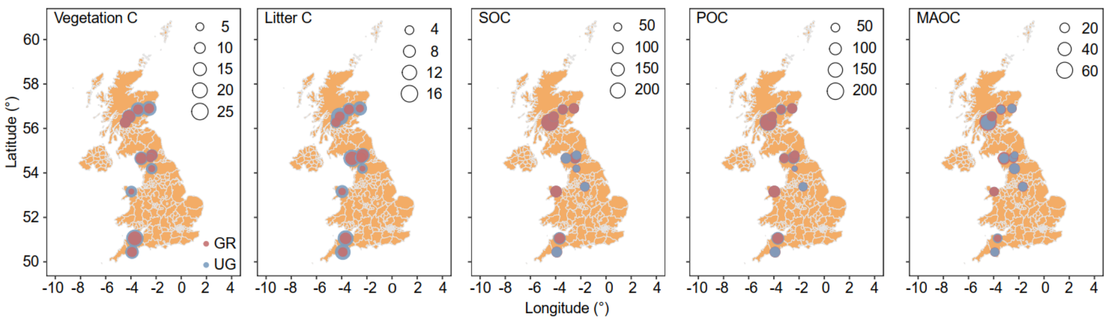

Fig. S1. Spatial distributions of the aboveground and belowground carbon (C) pools (Mg C ha^{-1}) across montane grasslands along an 800-kilometer gradient. SOC, soil organic C; POC, particulate organic C; MAOC, mineral-associated organic C.

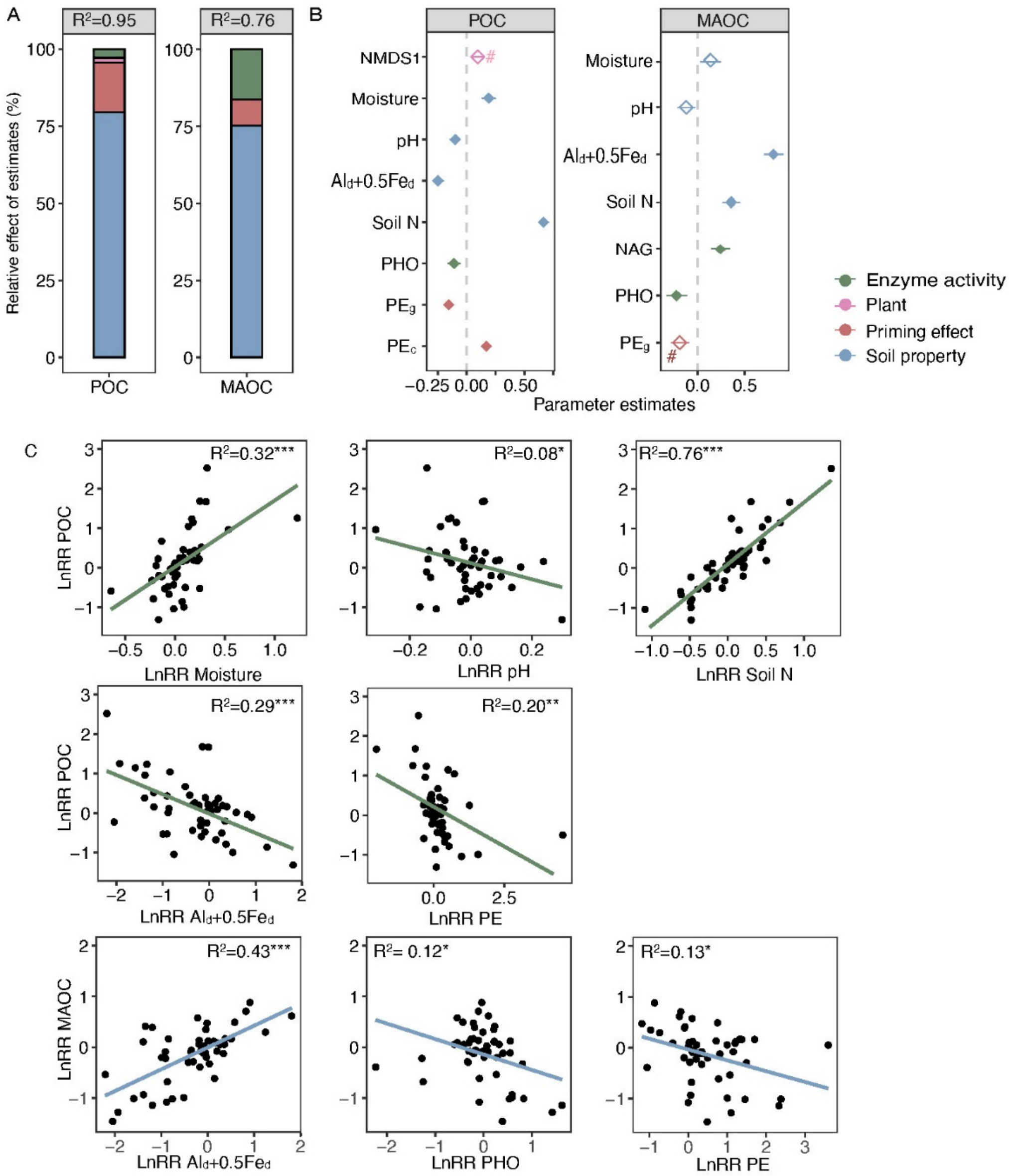

Fig. S2. Linking response ratio (LnRR) in abiotic and biotic factors with LnRR of particulate organic carbon (POC) and mineral-associated carbon (MAOC) associated with grazer exclusion. A, the relative importance of plant, enzyme, priming effect, and soil properties; **B,** average parameter estimates of model predictors, and associated standard error; **C,** linear relationships between predictors and carbon components based on a linear mixed model. The R^2 of the averaged model and the *P* value are shown as: $^{\#}P < 0.1$; $^{*}P < 0.05$; $^{**}P < 0.01$; $^{***}P < 0.001$. NMDS1, the first axis of Nonmetric multidimensional scaling (NMDS), shows the differences in the plant functional composition; $Al_d+0.5Fe_d$, weight-normalized contents of Fe_d and Al_d; GLC, β-glucosidase; NAG, N-acetyl glucosaminidase; PHO, phosphatase; POX, phenoloxidase; PER, peroxidase; PE_g, glucose-induced priming effect; PE_c, cellulose-induced priming effect.

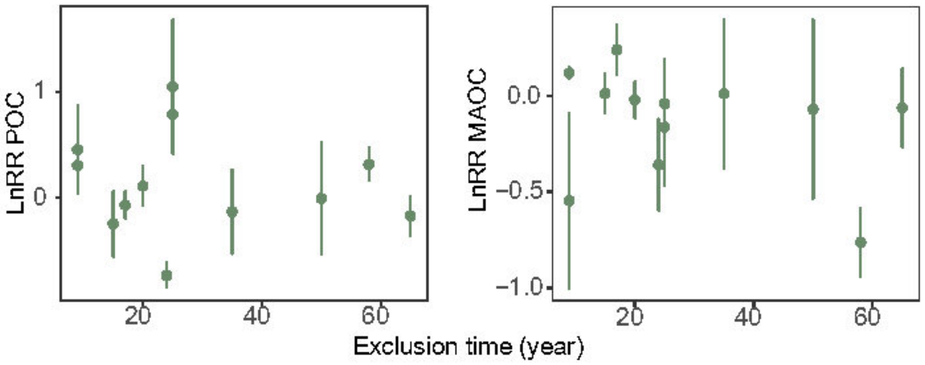

Fig. S3. Effect of the duration of grazer exclusion on differences in particulate organic carbon (POC) and mineral-associated organic carbon (MAOC). LnRR denotes the response ratio.

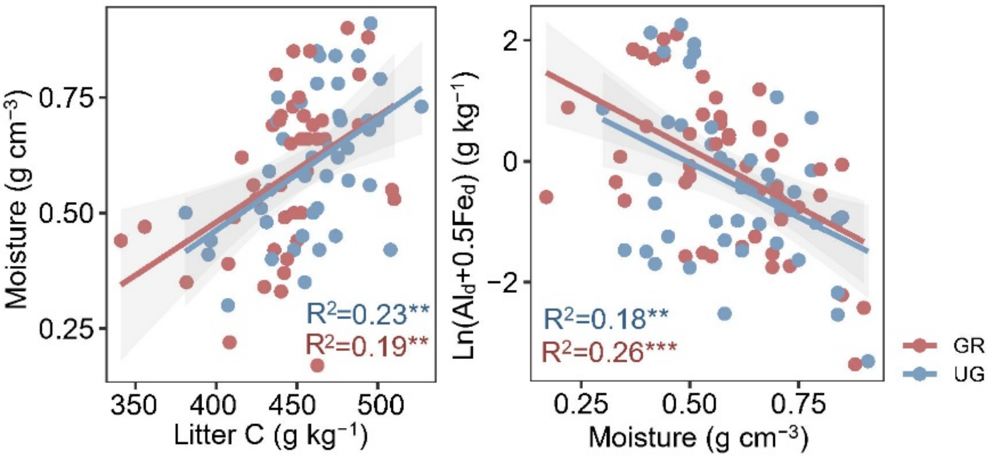

Fig. S4. Linear regressions between soil moisture and mineral content and litter C content. Grazing, GR; grazer exclusion, UG. The shaded areas represent the 95% confidence intervals. $^{**}P < 0.01$; $^{***}P < 0.001$. $Al_d+0.5Fe_d$, weight-normalized contents of Fe_d and Al_d.

Table S1. Soil textures and slopes of sampling plots across twelve study sites under ungrazed and grazed management. Linear mixed-effects models revealed no significant differences between treatments for either texture (P = 0.51) or slope (P = 0.57).

Site	Silt + clay content (%)		Slope (°)	
	Grazing	Grazer exclusion	Grazing	Grazer exclusion
BENL	22.00 ± 5.27	22.59 ±1.58	3.50 ± 0.87	3.25 ± 0.63
DALE	55.61 ± 5.99	55.96 ± 4.48	14.50 ± 4.17	14.00 ± 3.67
DART	19.44 ± 2.24	19.28 ± 2.03	8.00± 1.78	7.50 ± 1.44
EXMO	12.40 ± 1.82	17.29 ± 0.70	4.67 ± 1.45	6.33 ± 2.67
FING	25.90 ± 6.23	24.60 ± 7.61	11.75 ± 2.72	11.50 ± 2.72
LAKE	54.89 ± 4.85	50.43 ± 6.03	6.00 ± 1.58	6.50 ± 5.52
MOO1	31.46 ± 10.53	28.49 ± 6.37	3.75 ± 2.17	3.25 ± 1.89
MOO2	15.51 ± 2.19	19.76 ± 3.44	2.50 ± 1.26	1.50 ± 0.87
PEAK	28.83 ± 2.23	23.63 ± 2.02	7.17 ± 3.87	13.5 ± 0.43
SAUG	20.26 ± 4.88	20.06 ± 3.77	9.00 ± 0.41	8.50 ± 0.29
SHEE	33.61 ± 3.50	26.84 ± 4.59	8.50 ± 1.85	9.00 ± 2.04
SNOW	21.99 ± 1.64	25.22 ± 2.11	11.50 ± 1.19	8.50 ± 0.65
Overall	28.83 ± 2.25	27.89 ± 1.99	7.61 ± 0.82	8.04 ± 0.81
P value	0.51		0.57	

Sites include Ben Lawers (BENL), Dartmoor (DART), Exmoor (EXMO), Glen Flinglas (FING), Glensaugh (SAUG), Glenshee (SHEE), Lake District (LAKE), Moor House (MOO1), North Pennines (MOO2), Peak District (PEAK), Snowdonia (SNOW), and Yorkshire Dales (DALE).

Table S2. Linear mixed-effects models show the relationship between plant and soil properties in control plots and differences in POC and MAOC between grazed and ungrazed plots.

**Predictors**	**Response ratio of POC**		**Response ratio of MAOC**	
	**Slope**	***P* Value**	**Slope**	***P* Value**
Duration of grazing exclusion (year)	-3.72×10^{-3}	0.65	-4.19×10^{-3}	0.37
AM cover (%)	4.10×10^{-3}	0.22	9.24×10^{-4}	0.71
ErM cover (%)	-0.01	**0.03**	3.44×10^{-3}	0.36
Organic layer (cm)	-0.02	0.05	0.01	0.18
Aboveground biomass (kg m^{-2})	-0.11	0.37	0.03	0.74
Vegetation C (g kg^{-1})	-4.42×10^{-3}	0.33	-1.60×10^{-4}	0.96
Vegetation N (g kg^{-1})	-3.85×10^{-3}	0.94	-0.03	0.36
Vegetation C: N	-0.01	0.59	0.01	0.38
Litter biomass (kg m^{-2})	0.01	0.92	0.03	0.69
Litter C (g kg^{-1})	-0.01	0.18	-2.49×10^{-4}	0.93
Litter N (g kg^{-1})	-0.01	0.87	-0.01	0.78
Litter C: N	-0.01	0.58	3.00×10^{-3}	0.83
Moisture (g cm^{-3})	-2.00	**<0.01**	0.13	0.78
pH	0.44	**0.03**	0.02	0.87
Bulk density (g cm^{-3})	1.11	**<0.01**	-0.07	0.79
POC (g kg^{-1})	-2.74×10^{-3}	**<0.001**	1.85×10^{-4}	0.75
MAOC (g kg^{-1})	4.57×10^{-3}	0.33	-0.01	**<0.001**
Soil N (g kg^{-1})	-0.05	**<0.01**	-0.01	0.42
Fe_d (g kg^{-1})	0.07	0.09	0.01	0.69
Al_d (g kg^{-1})	0.76	**<0.01**	-0.01	0.94
Al_d+ $0.5Fe_d$ (g kg^{-1})	0.12	**0.04**	0.01	0.77

AM, arbuscular mycorrhizal species; ErM, ericoid mycorrhizal species; C, carbon; N, nitrogen; POC, particulate organic C; MAOC, mineral-associated organic C; $Al_d+0.5Fe_d$, weight-normalized contents of Fe_d and Al_d.

Table S3. Statistical power to detect the effect of grazer removal on soil C fractions at different effect sizes at a 0.05 significance level.

Variables	Cohen's d effect size		
	0.2 (Small effects)	0.5 (Medium effects)	0.8 (Large effects)
POC	28.8%	92.2%	100.0%
MAOC	31.8%	94.6%	100.0%
Aboveground biomass	28.8%	92.5%	100.0%
Litter biomass	32.4%	94.7%	100.0%

POC, particulate organic carbon; MAOC, mineral-associated organic carbon.
